# The e-health implementation toolkit: qualitative evaluation across four European countries

**DOI:** 10.1186/1748-5908-6-122

**Published:** 2011-11-19

**Authors:** Anne MacFarlane, Pauline Clerkin, Elizabeth Murray, David J Heaney, Mary Wakeling, Ulla-Maija Pesola, Eva Lindh Waterworth, Frank Larsen, Minna Makiniemi, Ilkka Winblad

**Affiliations:** 1 Graduate Entry Medical School, University of Limerick, Limerick, Ireland; 2 Discipline of General Practice, National University of Ireland, Galway, Galway, Ireland; 3 e-Health Unit, Department of Primary Care & Population Health, University College London, Upper Floor 3, Royal Free Hospital, Rowland Hill Street, London NW3 2PF, UK; 4 Centre for Rural Health, University of Aberdeen, Inverness, UK; 5 Department of Informatics, Umeå University, Umeå, Sweden; 6 Norwegian Centre for Integrated Care and Telemedicine, University Hospital of North Norway, Tromsø, Norway; 7 Oulu University Hospital, Northern Ostrobothnia Hospital District, Oulu, Finland; 8 Oulu University, Oulu, Finland

## Abstract

**Background:**

Implementation researchers have attempted to overcome the research-practice gap in e-health by developing tools that summarize and synthesize research evidence of factors that impede or facilitate implementation of innovation in healthcare settings. The e-Health Implementation Toolkit (e-HIT) is an example of such a tool that was designed within the context of the United Kingdom National Health Service to promote implementation of e-health services. Its utility in international settings is unknown.

**Methods:**

We conducted a qualitative evaluation of the e-HIT in use across four countries--Finland, Norway, Scotland, and Sweden. Data were generated using a combination of interview approaches (n = 22) to document e-HIT users' experiences of the tool to guide decision making about the selection of e-health pilot services and to monitor their progress over time.

**Results:**

e-HIT users evaluated the tool positively in terms of its scope to organize and enhance their critical thinking about their implementation work and, importantly, to facilitate discussion between those involved in that work. It was easy to use in either its paper- or web-based format, and its visual elements were positively received. There were some minor criticisms of the e-HIT with some suggestions for content changes and comments about its design as a generic tool (rather than specific to sites and e-health services). However, overall, e-HIT users considered it to be a highly workable tool that they found useful, which they would use again, and which they would recommend to other e-health implementers.

**Conclusion:**

The use of the e-HIT is feasible and acceptable in a range of international contexts by a range of professionals for a range of different e-health systems.

## Background

Healthcare systems across the developed world face shared challenges in terms of rising healthcare costs related to an aging population, increased prevalence of long-term conditions, and new treatments leading to improved survival [1]. A common strategy for addressing these challenges is the development of e-health, or the use of information and communication technology in healthcare, which is seen as having the potential to improve access to high-quality healthcare in a cost-effective fashion [2,3]. However, implementation of e-health initiatives is often difficult, with well-documented problems of delay, budget overspends, and occasional severely negative impacts on the quality and effectiveness of care [4-6]. These difficulties have continued, despite a considerable literature on implementing e-health systems, with a growing awareness of the importance of a socio-technical approach, *i.e.*, the importance of the inter-relation between technology and the social environment [7,8].

There are many possible reasons why implementation of e-health systems continues to be challenging despite the available literature. Some of these are likely to parallel those contributing to the gap between research findings in general and routine clinical care [9], including: a perceived lack of relevance of research to practitioner needs; responsible staff not having the time or inclination to read a large body of literature [10]; inadequacies in the existing research [11]; and the poor permeability of the managerial/research interface [12].

Implementation researchers have attempted to overcome this translational gap by developing tools that summarize and synthesize research evidence of factors that impede or facilitate implementation of innovation in healthcare settings. While still relatively rare [13], there is a growing body of such tools that are designed to promote implementation generally [14-17] and in the field of e-health specifically [13,18,19].

The e-Health Implementation Toolkit (e-HIT) is an example of a tool designed to promote implementation of e-health services and, like other tools, it was designed to present evidence about e-health implementation in a format that could easily be digested and used by staff considering or planning an implementation [19]. It was developed by combining three sources of information: data from a systematic review of reviews of implementation of e-health; qualitative data derived from interviews with senior staff responsible for an e-health implementation in the UK; and the Normalization Process Theory (NPT). The NPT is a sociological theory that explains why some new technologies or practices become part of routine practice, and some do not. It focuses on the work individuals and groups need to undertake for a technology or practice to be implemented and become integrated into everyday use [20]. It thus provides a theoretical framework for understanding the important inter-relationship between technology and the social environment, and has been used to develop other theory-driven implementation tools and frameworks [21,22].

The initial formative evaluation of the e-HIT noted that it was unclear whether the toolkit would be useful outside the context in which it was initially developed, *i.e.*, the United Kingdom (UK) National Health Service [19]. In this study we aimed to explore the utility of the e-HIT from an international user perspective. Specific objectives were to: describe the ways in which the e-HIT was used in different international contexts; evaluate users' views about the workability and usefulness of the e-HIT; and suggest improvements or modifications to the e-HIT.

## Methods

### Context

**Figure 1:**
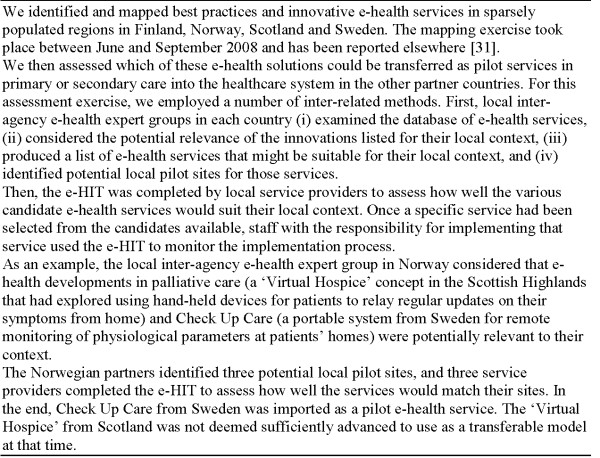
**E-health practices across the Northern Periphery Area: Project summary **[31].

The context for this study was a large project funded by the European Union (EU). The aim was to enhance the provision and accessibility of health services in sparsely populated areas of Europe by developing and implementing innovative e-health services and promoting transfer of the best e-health practices across the Northern Periphery Area. The Northern Periphery Area extends across sparsely populated areas of Scotland, Norway and Sweden, most of Finland, and all of Greenland, Iceland, and the Faroe Islands (Northern Periphery Programme website is http://www.northernperiphery.eu). In this project, we focused on sparsely populated northern periphery regions in Finland, Norway, Scotland, and Sweden [23]. Figure 1 provides a summary of the project. It describes a mapping exercise of e-health services across the four countries of interest from which a database of e-health services was compiled. E-health services were selected from the database for transfer from one partner country to another partner country as pilot e-health services.

The e-HIT was used as an intervention to inform decisions about which e-health pilot services to implement at which sites, and also to monitor the implementation work of these selected pilot services. The use of the e-HIT in this project provides an excellent opportunity for an evaluation of this newly developed tool from an international user perspective. The service providers in the study were facing exactly the tasks the e-HIT was designed for, *i.e.*, choosing whether or not to proceed with implementing a given e-health initiative, and then monitoring the implementation process over time. Furthermore, three of the four implementation projects were based outside the context in which the e-HIT was initially developed.

### Intervention

**Figure 2:**
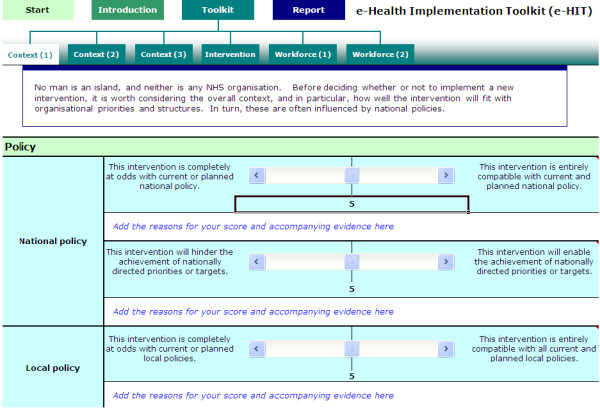
**The e-Health Implementation ToolKit**.

The development and formative evaluation of the e-HIT has been described elsewhere [19]. The goal of the e-HIT was to act as a sensitizing agent to enable senior staff to think through the challenges and problems likely to arise when implementing an e-health initiative. Advice on how to use the toolkit included getting staff from all the different professional groups likely to be affected by the implementation to complete the e-HIT and compare and discuss results. It was not designed as a 'tick-box' tool, and was intended to provide a structure to promote critical thinking, not replace it. The e-HIT was a freely downloadable toolkit, in the format of an Excel spreadsheet http://www.ucl.ac.uk/silva/pcph/research-groups-themes/e-health/resources, also see Figure 2). There were three sections: an introduction for novice users; exemplar case studies; and the toolkit itself. The toolkit consisted of six pages, with three or four statements on each page. Each statement was phrased as both an extreme negative and an extreme positive statement (*e.g.*, the proposed e-health initiative will disrupt patient-professional interactions/the proposed initiative will facilitate patient-professional interactions). Under each statement was a sliding bar with a scale from 0 to 10. Users were asked to consider each statement in terms of the specific initiative under consideration, and for the context in which implementation was planned. A box for free text was provided where users could enter the reasons for the score given, and any comments.

The statements were grouped into three main areas: context (national and local policy, leadership, resources); the intervention itself (usability, fitness for purpose); and the workforce (impact on workload, workflow, division of labour, training requirements, power relationships, allocation of responsibility and accountability). After users had completed each of the statements on the six pages, the toolkit analyzed their input and provided a report. The aim of the report was to highlight issues that were likely to go relatively smoothly during the implementation, and alert the user to areas that needed more attention. Text provided with the report emphasized again that the aim was to provide a tool to promote and structure critical thinking, not to act as a 'tick-box' approach.

### Sampling and recruitment

Participants for this qualitative evaluation were healthcare managers, clinicians, and practitioners who used the e-HIT in the selection of pilot implementation e-health services in Finland, Norway, Scotland, and Sweden (Figure 1).

Following the principles of purposive sampling [24], we invited these personnel to participate in this evaluation. We asked project partners to act as a link between the Ireland-based researchers (AMacF and PC), who were leading the evaluation, and potential participants in each country. For instance, our Scottish partner sent information about the evaluation, an invitation to participate, and contact details of the Ireland-based researchers to Scottish healthcare managers, clinicians, and practitioners working in the project who had used the e-HIT. Those who agreed to be interviewed consented to their contact details being given to the Ireland-based researchers who then proceeded with data collection.

We undertook two recruitment drives. The first, in October 2009, was focused around the recruitment of healthcare managers, clinicians and practitioners who had used the e-HIT to guide the selection of pilot e-health services. The response to this first round of recruitment was slow and we identified that the main reasons for non-participation were: lack of time; discomfort among Finnish, Norwegian, and Swedish participants about conducting interviews in the English language with the researcher based in Ireland; and concerns that they would not remember sufficient details of the e-HIT to warrant their participation in the research.

We adopted a different approach for the second round of recruitment, which took place between June and October 2010, and was focused around the recruitment of healthcare managers, clinicians, and practitioners who had used the e-HIT to monitor the implementation of established pilot e-health services. We informed potential participants that they could, if they wished, be interviewed in their own language with the project researcher in their own country. We also planned to conduct the interviews with e-HIT users closer in time to their use of the tool to monitor the implementation of the established pilot e-health services. This improved recruitment rates considerably (Table 1).

**Table 1:** Number of e-HIT users and interviews per country for qualitative evaluation of e-HIT

Country	e-HIT users for selection of pilot e-health services n = 32	Interviews re use of e-HIT for selection n = 8	e-HIT users for monitoring implementation of pilot e-health services n = 18	Interviews re use of e-HIT for monitoring n = 14
**Finland**	18	4	8	8

**Norway**	3	1	3	1

**Scotland**	3	2	4	4

**Sweden**	8	1	3	1

### Data collection and analysis

Interviews provide a key way to document people's experiences, providing a unique access to the lived world of participants, who in their own words describe their activities, experiences and opinions [25]. They can be conducted face-to-face or by telephone, and one-to-one or as a group [25,26].

We used a combination of interview methods and types in this research. This was primarily for pragmatic reasons--we had to address the geographical challenges of transnational research and accommodate our participants' time schedules and their preferences around being interviewed in English or their own language. Some interviews were telephone interviews between the Ireland-based researcher (PC) and participants. These were conducted in English or in the participant's own language, in which case the local project researcher acted as interpreter. Others were face-to-face interviews conducted in participants' own language between themselves and the project researcher in their own country. These interviews were then translated by the project researcher involved so that English-speaking researchers could engage with the full data set for analysis.

In keeping with the iterative nature of qualitative research, we developed an interview topic guide based on our research aims and objectives, and we modified this as necessary based on field notes from data collection, reflective discussions at research meetings, and emerging findings from the initial analysis of collected data. Further questions were also added to the topic guide to make it more culturally specific, on the suggestion of one of the project partners. For example, questions were included on whether the e-HIT items suited the healthcare structure of the country involved and the way the service provision unit involved in the implementation of the pilot e-health service in question operates.

The topic guide used in the first round of interviews focused on describing the way in which the e-HIT was used for decision making about pilot services as well as its usability and workability, its relevance, accuracy, and comprehensiveness for the planned implementation work.

The topic guide used in the second round of interviews also covered issues of usability, workability, relevance, accuracy, and comprehensiveness, but with a focus on the ongoing implementation work, by exploring ways in which the e-HIT informed participants' assessment of that work.

We transcribed data for analysis and followed the principles of thematic analysis according to the precepts of grounded theory [27].

Social scientists AMacF and PC led the analysis and shared the emergent analysis with clinician EM. This brought an interdisciplinary dimension to the analysis, a process known to enhance reliability [28]. This discursive and reflexive inter-disciplinary sharing was complemented by a further layer of discussion and reflexivity about the data and their interpretation with members of the project team in each country. This was conducted by e-mail and telephone and ensured that project partners who had been involved in generating data in their own countries could inform the analysis of data, and check that any country-specific perspectives were presented faithfully and fully understood by the researchers leading the analysis. This was important for the authenticity of the analysis process.

## Results

Table 1 shows the number of e-HIT users per country and the number recruited for this qualitative evaluation of the e-HIT.

The total number of e-HIT users in the study is 50. There were 22 interviews conducted for this qualitative evaluation--eight e-HIT users were interviewed about their use of the tool to select pilot e-health services and 14 were interviewed about their use of the tool to monitor their implementation. The total sample size is 14 because eight participants completed the e-HIT twice and were interviewed twice.

Table 2 provides information on the professional background of interview participants and an overview of our data collection methods in each country.

**Table 2:** Overview of interview participants' professional backgrounds and data collection methods per country

Country	Selecting pilot e-health services Participants n = 8	Monitoring pilot e-health implementation services Participants n = 14
Finland	1 × group telephone interview (n = 4) with PC^ interpreted by MM • 1 × e-Health Research Project Manager • 1 × Medical Doctor • 1 × Director of Health Services • 1 × Nurse, wound specialist	1 × group face-to-face interview (n = 3) with MM • 1 × Product Testing Specialist • 1 × Chief Physician • 1 × Public Health Nurse 1 × group face-to-face interview (n = 2) with MM • 1 × Dermatologist • 1 × Nurse, wound specialist 1 × group telephone interview (n = 3) with MM • 1 × Chief Physician • 1 × Nurse, wound specialist • 1 × Speech Therapist

Norway	1 × individual face-to-face interview with FL • 1 × Nurse coordinator of the reorganization of GP services in 4 municipalities	1 × individual telephone interview with FL • 1 × Nurse, nursing home setting

Scotland	2 × individual telephone interviews with PC • 1 × Speech Therapist • 1 × Charge Nurse	4 × individual face-to-face interviews with MW • 1 × Speech Therapist • 1 × Charge Nurse • 1 × Renal Consultant • 1 × Clinical Ward Manager

Sweden	1 × individual face-to-face interview with UMP • 1 × Opthamology Unit Manager (nurse)	1 × individual face-to-face interview with UMP • 1 × Opthamology Unit Manager (nurse)

^initials shown here are of researchers/authors who contributed to data collection, *e.g.*, PC is P. Clerkin, MM is M. Makiniemi, and so on.

Throughout this results section, quotes are coded by the respondent's profession (except where the data represent a collective view from a group interview) and country as follows:

CN (Charge Nurse), CWM (Clinical Ward Manager), DT (Dermatologist), OUM-N (Ophthalmology Unit Manager (nurse)), PTS (Product Testing Specialist), RC (Renal Consultant), ST (Speech Therapist), F (Finland), N (Norway), Sc (Scotland), and Sw (Sweden).

### Use of the e-HIT across project sites

Most participants used the online version of the e-HIT. Some used a paper version because the online version was deemed to be unsuited to the healthcare structure of that country. Using a paper version allowed them to make minor modifications to questions so that they were more culturally specific. As an example, in Finland primary healthcare is provided by municipalities, which are independent decision makers, small in nature, and based in peripheral sites. Specialized healthcare is provided in a separate organization by federations of municipalities. This means that nurses work more independently than in many other countries. As eHIT users, they had difficulties differentiating between national, regional, or local policies. For this reason, questions were modified in the paper version by a member of the project team in Finland to reflect this. Participants who worked with this modified version felt that it suited the structure of their healthcare system and their unit:

'Yes they say they [questions] fit perfectly...after his [project researcher's] modification' (F).

Most participants completed the e-HIT on their own, although a few were given assistance by project researchers when using the e-HIT toolkit:

'I used the toolkit together with [name of project researcher] at our department (ophthalmology unit). I used her laptop and she explained to me how it works, where to click and also what some of the questions mean. Then I just went through the questions' (OUM-N-Sw).

Overall, participants said that it was best for them to use the e-HIT alone and when they had sufficient time to reflect on the content, their answers, and comments.

### Views about the workability of the e-HIT

All participants reported that the e-HIT toolkit was easy to use. Most relied on the instructions in the toolkit, which they described as clear, straightforward, and user friendly. One participant did report some apprehension before using it but explained that it was, in fact, very user friendly:

'Sometimes when you go in for these things ... because perhaps we're not so used to using technology, you kind of think 'is this going to work?' or 'is it going to confuse me?' and it [e-HIT] was very easy and just took me through it step by step and there were lots of opportunities.... It gave me opportunities to go back if I needed to go back. I was impressed with how easy it was to use' (ST-Sc).

'The tool itself is fairly straightforward. Yes. Simple sort of scale 0 to 10 with some space for additional comments. Quite a straightforward tool' (CN-Sc).

Some participants did report difficulties with the toolkit, but they thought that this may have been due their own personal, computer, or system errors:

'Sometimes it wouldn't save bits and pieces of your comments and you had to go back, but some of it was maybe my user error, but obviously if there was some user error it's maybe not quite as intuitive' (CWM-Sc).

'It took a very very long time for each page to come up, uhm, a very, very, very, very long time, and I think if we had had the paper version we could probably have filled it in much more quickly .... I think it was more the system that failed then the tool itself. I found it quite a useful and easy tool to work with' (ST-Sc).

Some others reported difficulties with the 'slider' feature of the online toolkit:

'It was difficult using the slider, so we had to type in the, em, sort of score, em, apart from that the only real problem was the fact that the pages took so long to come up' (ST-Sc).

Overall, participants thought that the interface of the online e-HIT was 'quite well laid out, quite logical' (CN-Sc WP3) and considered that the 'slider' feature, which allows grading of toolkit items numerically, was a positive feature of the toolkit:

'If I remember correctly it was just a case of putting in a score of one to ten for each point and writing in some comments beside it and yeah it was very straightforward to fill in yeah.... I mean the fact that I filled it in in one session and I didn't have to ring or email anyone for advice so that speaks for itself' (CN-Sc).

'I actually liked the fact that you could grade it numerically but also qualify that. I like the fact that there were two... you know the two extremes were there for you. No it was quite comprehensive in that way' (ST-Sc).

Interestingly, the visual element of the e-HIT summary was seen as a positive feature that helped accentuate the respective strengths and potential weaknesses of the project:

'I like the fact that you can just go right through and come up with the summary and the scores at the end and that it's presented visually, not just the statistics though the statistics are there as well but the visual kinda just confronts you and it's not a surprise because you've gone through the process already but just seeing it presented in that way kind of highlights the strengths and the weaknesses and perhaps the obstacles that need to be overcome for you to take something forward' (ST-Sc).

It was notable that most of the difficulties mentioned about using the toolkit were related to the relevance of some questions to the specific project that participants were thinking about, rather than the usability of the toolkit *per se*:

'I seem to remember there was the odd question that was awkward to answer and difficult to score but the vast majority of them fitted into the context of what we're doing quite well and I don't remember having any particular problems with it' (CN-Sc).

### Usefulness of the e-HIT for decision making

Responses varied slightly in terms of whether using the e-HIT toolkit helped with decision making. One of the Scottish participants and all the Finnish service planners reported that they had already made their decision about which project to implement before they used it, but that using the e-HIT confirmed their decisions:

'Initially they had made the decision already to pilot the services and it didn't really help or offer a lot of help in the decision-making process but it recorded their earlier thinking. They are saying that it recorded their earlier decision and confirmed what they had been thinking already' (F).

However, there was consensus among all users that using the e-HIT helped to quantify and qualify the finer details and potential problems as well as breaking the implementation process down logically into areas that may prove more challenging, for example, issues relating to the broader context such as resource and workforce issues:

'It did make me look at it logically. It went through the contexts and resources and also the staff that would be using it because ... there's two or three of you that are enthusiastic about something and it just makes you look at the broader context and the impact on other services and also in terms of the resources and financially' (ST-Sc).

'I mean scores where we'd actually identified potential problems that we then needed to .... helped us identify where we could identify potential snagging problems with the project. For example, I'm just running through the actual copy that I filled in. All the ones that got high scores there obviously wasn't going to be a problem but some of the ones that got lower scores for example resources, big financial challenges in the project which I mean that's quite an obvious one. But another one for me that I thought was important...I mean for me I thought it was important that we didn't unduly increase staff workload. And just making reasoned awareness that with any new technology there was likely to be snagging issues. Just looking at them... the impact on workflow, em, education and training was a big one as I identified. For any project like this you have to put the right training in place or it's just gonna fall flat on its face' (CN-Sc).

This was similar for those who completed the toolkit in paper format, in that they too felt that using it helped solidify their thoughts and forced them to think of potential implementation issues:

'They also wanted to add that it broadened the view of the service because it provided additional aspects of information and it... broadened horizons and supported their earlier thinking' (F).

Service users reported that this major benefit of the e-HIT--the fact that it enabled them to break down the overall view of the potential e-health pilot service into a clear, logical, and standardized format with attention to positives and negatives--led to another major benefit of the tool: it made it much easier for them to explain and discuss the project with other people:

'Because it made me look at it logically it also means that if I had to come and explain the project to somebody else then I've done that thinking and whilst it might not save any money just now the whole plan is that it will save money in the future' (ST-Sc).

Similarly,

'Where I think the toolkit was useful was em... where you've got multiple people dealing with a project communication is the issue and what seems obvious to me isn't necessarily obvious to someone else. So I think where the toolkit was useful was to put all that down in a standardized format that other people could look at, question if they wanted more detail, and either agree or disagree with so that we're all sort of singing from the same sheet, so to speak' (CN-Sc).

### Usefulness of the e-HIT to monitor the implementation of pilot e-health services

Participants found that the e-HIT toolkit was very useful for monitoring the implementation of pilot e-health services, particularly for comparing imagined implementation issues (when using the e-HIT to inform decision making) and experienced implementation issues:

'I found it a useful exercise for me to reflect on what had happened and a useful exercise to compare what I'd thought at the outset with my thinking now' (ST-Sc).

Similar to those responses about using e-HIT for decision making, participants reported that the e-HIT was comprehensive and that it was a tool that facilitated the formalization of thoughts about implementation process:

'I think it's actually quite clever the way it does it, it does seem to cover most of the issues that you would, that you, well, we have come across' (RC-Sc).

'I suppose it was very formal terminology and as I was going through it, some of it I thought, oh, I didn't really give that much thought when we were implementing it, or I didn't give, I didn't give it em... I suppose very formal thought on these elements but then when you read it you then reflect and think well, yes, that was an aspect of it, if you have to formalize it and put it into words that possibly some of these things were aspects of it that I maybe wasn't overly conscious of' (CWM-Sc).

There were some critical comments, for example that the language was at times a bit 'highbrow' and others didn't feel that all items were relevant to everyday practice. Overall, however, it was considered helpful for monitoring implementation of the pilot e-health services and, as before, particularly where a team of people are involved with the implementation work.

### Recommendations for changing the e-HIT

Participants were very positive about the content of the e-HIT. They felt that the questions were accurate, relevant, and addressed a comprehensive range of important issues for e-health implementation:

'They feel that the questions were relevant and provided more information so they could look at the service from different angles' (F).

'I think they were relevant' (ST-F)

'They feel that this was quite comprehensive with lots of questions and they added that some of the questions expanded the point of view - that they got a better understanding' (F).

However, there were some suggested changes to the toolkit. The Finnish project partner suggested incorporating a SWOT (strengths, weaknesses, opportunities, and threats) analysis into the toolkit, and also comparing the results of both exercises. Indeed, in Finland the interviews were complemented by: an analysis of the quality of the room where the application was to be used including assessment of the quality of the IT connections; and SWOT analyses in order to have a general point of view of strengths, weaknesses, opportunities, and threats of the application.

The staff in the Finnish healthcare centres thought that there were questions in the e-HIT that are more relevant to a higher administrative level than to employees at the level of practical service delivery (although this is the stated target audience for the toolkit). They also thought that there could have been questions on technical functionality.

The Swedish service provider thought that a more practical and specific aspect to the toolkit would make it more useful:

'The toolkit seems to be of a very general nature, it doesn't really say anything about EyeMo [the pilot e-health service implemented in Sweden]. I would like it to be more practical and more detailed so that it would include something about EyeMo that we are using but maybe it is too difficult to make something like that. The practical side of e-health solutions is missing from the toolkit. Some of the questions leave a lot of room for interpretation and there are many questions in one question and then it becomes very hard for me to answer because I might not necessarily give the same answer to those questions' (OUM-N-Sw).

The non-specificity of the toolkit was also mentioned by the service providers from Finland and Scotland:

'As I said in the beginning, the questions should be more targeted [to a specific e-health service]' (DT-F).

'Not a lot was said about the equipment, more could have been asked about them, one would have expected more questions on problems associated with the equipment' (F).

'It's obviously a generic tool. Could it be tailored for individual projects where some questions are taken out and the ability to put in some extra questions and maybe to add in some extra detail? Em... that would be, it's a minor change and, as I say, on the whole I think it worked well' (CN-Sc).

Norwegian participants mentioned this issue as well and explained that they had to try to find an empirical equivalent, something that corresponds to the concept in the real world, in Norway, especially when we are talking about context variables. At the same time, they explained that the positive thing about a generic questionnaire is, of course, that it fits a lot of services, organizations, and national policies and therefore is of international relevance.

The service providers from Finland had some suggestions around the scales, although they acknowledged that problems they may have had were likely to have been culturally influenced by the use of a school grades scale system, which differed from the scales used in the e-HIT:

'I think evaluation would be easier if the scale was a little smaller, perhaps from one to five or I don't know, perhaps even from one to three' (ST-F).

'This evaluation scale takes one's mind into the school world and school grades, and there three or four are not used, mostly grades are around from six, seven, eight, or even nine. Therefore, our preconceptions will not allow full use of the scale, our educational background guides or colours our use of it' (PTS-F).

The Scottish service providers suggested that a space for general comments and overall feelings would be useful as well as suggesting that an element for differentiation between user roles would help:

'I'm not aware there was a space for that, just to have general comments at the end, a summary of your overall feelings that you perhaps could summarize' (CWM-Sc).

'It doesn't ask who you are...what part you played in the project. But I don't know whether that's intentional that you might not want to know the discipline or who the person is [because] you might think that would sway the results but you may want, would you not want to know that? What one person in the multi-disciplinary team thinks [because] their opinions may be very different to what somebody else's role is in it' (CWM-Sc).

Finally, some participants were confused about which contextual level to think about when completing the e-HIT. Participants in Norway commented that it was difficult to know whether to respond to questions about 'organization' because e-health services involve co-operation between organizations located in different sites. A Swedish participant noted some confusion as to whether to think about e-health services in terms of the whole country or local council:

'Yes but well it's more like sometimes I feel it is difficult to know how to think if I should be thinking of this unit [ophthalmoloy] or what can I say the whole country or this county council [county council of Västerbotten] or the entire council or just this unit or the entire ophthalmology care then I would actually like to give different answers depending on that point of view if I was thinking of our unit or the entire county council' (OUM-N-Sw).

Notwithstanding these suggested changes to the e-HIT, the majority of participants (n = 10/14) said that they would definitely recommend using the e-HIT to others. This was the case both with those who had used it online and in paper format:

'Yes absolutely. I would certainly recommend it. As I said it takes you through quite a logical objective thinking process. It's up to them to choose it but I would certainly recommend it.' (ST-Sc).

'Yeah I think it's a useful tool. Particularly when you do a sort of pre- and post-project one as we've done and it's quite interesting to look back at your initial comments and make comparisons' (CN-Sc).

## Discussion

A large EU project across sparsely populated regions in four countries presented an ideal opportunity for evaluation of the e-HIT in use in international settings and, thus, to address an identified gap in knowledge about the utility of the e-HIT in settings other than the one in which it was developed (*i.e*., the UK National Health Service).

The present research involved a qualitative evaluation of eHIT users' experiences of using the tool to make decisions about which e-health services to implement in their local settings and to monitor the implementation work over time.

### Summary of findings and relationship with published literature

The e-HIT is part of a growing set of tools designed by researchers to synthesize and summarize research evidence about implementation issues and to present information about factors that promote and impede implementation processes [13-18]. While it is positive to see published accounts of the development of such tools, it is important that research considers their utility from a user perspective and explores this issue across healthcare systems [19]. To our knowledge, evaluation of users' experiences of using implementation tools is rare, and therefore our study makes an important contribution to the literature in this regard.

Overall the e-HIT has been evaluated positively in terms of its scope to organize and enhance critical thinking about implementation work and, importantly, to facilitate discussion between those involved in that work. Because e-health solutions are cross-border (units, departments, organizations) services, it is very positive to understand the ways in which the e-HIT allowed different implementers at different locations who were involved in the same project to see and to share their assessments of the work involved.

These findings indicate that the tool meets its goal, which is to act as a sensitizing agent to enable staff to think through the challenges and problems likely to arise when implementing an e-health initiative and to promote critical thinking [19].

Like other tools [13], the e-HIT was relatively quick to use but, importantly, there was no evidence of participants using the e-HIT as a 'tick-box' tool or in a rigid way. Instead, there are accounts of users critically thinking about the relevance of the questions for their context and the service with which they were concerned, and also of users modifying the tool or the way in which they used it to suit their own purposes, *e.g.*, changing items to make them more culturally specific and employing other strategies as well as the e-HIT to inform decision making about the selection of e-health services.

Wen *et al. *[13] used exclusive descriptions rather than Likert scale responses for their Readiness for Implementation tool on the basis that it is better to 'force' respondents to choose an answer. However, our data indicate that the e-HIT users liked the Likert scale element and the sliding scoring system that it offered them.

In terms of its workability, online and paper-based versions work equally well, and visual features (*i.e.*, e-HIT scales and summary) are appealing and user friendly. There were some reported technical difficulties (*e.g.*, slow loading of the toolkit and problems using the slider) but these difficulties are likely to be minimal if the toolkit is saved when downloaded rather than simply 'opened' online.

There were critical comments about the generic nature of the toolkit. However, it is unrealistic to expect that a toolkit could be both simple and brief enough to be usable in the way participants reported it to be, and also specific to their exact contexts. Moreover, we know from this analysis that it was relatively easy to make minor changes to the toolkit items to ensure that they are intervention- and/or country-specific.

Finally, while there were some recommended changes to the content, overall, participants said that the toolkit was useful, they would use it again, and would recommend it for use to other e-health implementers, which indicates that it has resonance and utility across international settings.

### Methodological strengths and limitations

This is a qualitative study that involved evaluation of the e-HIT in a range of e-health systems across four international sites. We did not employ an explicit theoretical framework to inform our evaluation of the e-HIT and, with hindsight, it would have been good to do so. Given our interest in issues of usability and workability of the e-HIT, we could have employed the NPT as our theoretical framework. However, at the same time, the NPT had been used to develop the e-HIT and, in order to be open to finding problems with the e-HIT, it would not have been a suitable theoretical framework for the evaluation.

Despite recruitment challenges and problems with language differences between the lead researchers in Ireland and participants in the other sites, we achieved a good sample size that allowed us to saturate understanding of the issues under investigation [29].

There was a pragmatic element to our fieldwork but all interviews conducted for this evaluation had the shared value of documenting participants' experiences and giving them an opportunity to describe their activities, experiences, and opinions in their own words [25]. There may have been some social desirability [30] in participants' reports of their experiences. However, during data collection we encouraged participants to express positive and negative aspects of their experiences, particularly by asking them to consider how the toolkit could be improved. This provided an explicit opportunity for participants to be critical about their experiences, which we have presented in this paper.

To enhance quality and rigour of the analysis process, we were committed to important inter-disciplinary and international dialogue about the data and their interpretation, which is important in cross-cultural research [28,30]. This dialogue led to discussions about, and questioning of, our data. This allowed us to elaborate and refine the presentation of results with specific examples to concretize findings, and to consider evidence of contrasting views around a particular issue (*e.g.*, the positive and negative implications of the e-HIT as a generic rather than a specific tool), which provided a more 'rounded' analysis.

## Conclusion

The use of the e-HIT toolkit is feasible and acceptable in a range of international contexts by a range of professionals for a range of different e-health systems. Users have to accept the need to do a bit of local adaptation and thinking about how best to use the toolkit--as they would before applying any research finding to their own situation. This probably needs stressing more in the instructions. In view of the need to get multiple users to complete and compare their entries, the toolkit does need web-enabling--this would also avoid any technical problems with slow page loading. Further work is merited to determine actual impact. It would be valuable to conduct a qualitative process evaluation of the e-HIT in use during decision-making processes to monitor and elucidate the way in which the toolkit is used (on its own or in conjunction with other methods) to inform decisions about implementation work. Furthermore, it would be valuable to design an evaluation of the e-HIT as a toolkit itself to support implementation of e-health systems. This would require a theoretically informed, prospective evaluation of the toolkit in use to examine if it can guide implementation work and/or anticipate implementation outcomes. This is an important area for further research and, based on our thoughts above about the scope to use NPT in this study (or not), there are interesting questions about which theoretical framework may be used to inform such research.

## Competing interests

EM led the development of the e-HIT in a previous research project. The authors declare that they have no other competing interests.

## Authors' contributions

AMacF led the use and qualitative evaluation of the e-Health Implementation Toolkit in this project and the write up of this manuscript. PC contributed to data collection, analysis and write up. EM contributed to study design, data analysis and write up. MM, IW, MW, FL, and UMP contributed to data collection and data analysis. DH and EW contributed to data analysis. All authors read and approved the final manuscript.
